# Strengthening Community Networks for Vital Event Reporting: Community-Based Reporting of Vital Events in Rural Mali

**DOI:** 10.1371/journal.pone.0132164

**Published:** 2015-11-25

**Authors:** Melinda K. Munos, Alain K. Koffi, Hamadoun Sangho, Mariam Guindo Traoré, Masseli Diakité, Romesh Silva, Mali RMM Working Group

**Affiliations:** 1 Department of International Health, Johns Hopkins Bloomberg School of Public Health, Baltimore, Maryland, United States of America; 2 Centre de Recherche, d’Etudes et de Documentation pour la Survie de l’Enfant, Bamako, Mali; University College London, UNITED KINGDOM

## Abstract

**Background:**

Like many developing countries, Mali has few sources of mortality data. High quality mortality estimates are available from household surveys, such as the demographic and health surveys (DHS), approximately every five years, making it difficult to track progress in reducing mortality. The Rapid Mortality Monitoring (RMM) project in Mali aimed to address this issue by testing a community-based approach to measuring under-five mortality on a yearly basis.

**Methods and Findings:**

Seventy-eight community-based workers (relais) were identified in 20 villages comprising approximately 5,300 households. The relais reported pregnancies, births, and under-five deaths from July, 2012 to November, 2013. Data were double-entered, reconciled, cleaned, and analyzed monthly. In November-December 2013, we administered a full pregnancy history (FPH) to women of reproductive age in a census of the households in the project villages. We assessed the completeness of the counts of births and deaths, and the validity of under-five, infant, and neonatal mortality rates from the community-based method against the retrospective FPH for two rolling twelve-month periods. Monthly reporting by relais was high, with reports on pregnancies, births, and deaths consistently provided from all 78 relais catchment areas. Relais reported 1,660 live births and 276 under-five deaths from July, 2012 to November, 2013. The under-five mortality rate calculated from the relais data was similar to that estimated using the validation survey, where the overall ratios of the community-based to FPH-based mortality rates for the reporting periods were 100.4 (95% CI: 80.4, 120.5) and 100.8 (95% CI: 79.5, 122.0).

**Conclusions:**

On a small scale, the community-based method in Mali produced estimates of annualized under-five mortality rates that were consistent with those obtained from a FPH. The community-based method should be considered for scale-up in Mali, with appropriate measures to ensure community engagement, data quality, and cross-validation with comparable FPHs.

## Introduction

At international level, and within most developing countries, programs are being implemented with the goal of reducing mortality in one of the most vulnerable population groups—children under five. Understanding whether these programs are able to reduce under-five mortality (and why) is of pressing concern for donors, countries, program implementers, and researchers. Unfortunately, answering this question, particularly over short time periods, is complicated by the challenges in measuring mortality change. In most developing countries, civil registration is incomplete, and the main sources of mortality estimates are the health management information system (HMIS) and household surveys such as the Demographic and Health Surveys (DHS) program [1]. HMIS generally only take into account deaths in health facilities, and do not include those at home (that is to say, in the community, where most deaths occur). DHS take place approximately every five years, and between two surveys, it is not possible to know whether or how mortality is changing. Furthermore, household surveys with full birth histories, such as the DHS, report estimates for a five-year period before the survey, and do not have sample sizes large enough to allow monitoring of mortality changes in “real time” (i.e., every 12 months).

To address this problem, we sought to develop and test community-based approaches to “real-time” (yearly) mortality measurement that could be built on a country’s existing health system or civil registration system, in five countries in sub-Saharan Africa through the Real-time Mortality Monitoring project (RMM).

This paper reports on the implementation and validation of the community-based approach to recording vital events in Mali—a large, land-locked country in West Africa with a relatively small population (14.5 million in 2009) [2], and very high fertility (total fertility rate of 6.1 in 2012) [3]. Although under-five mortality has decreased over the past two decades, almost 13% of live-born children still die before their fifth birthday [4]. Like most countries, Mali relies primarily on surveys such as DHS, which have taken place at intervals of six to seven years, to track changes in under-five mortality.

The objectives of RMM in Mali were to implement a community-based RMM method that builds on the existing structures of volunteers and health systems, and to assess the validity of this method in measuring under-five mortality over 12-month periods.

## Methods

### Setting and selection of RMM area

**Fig 1:**
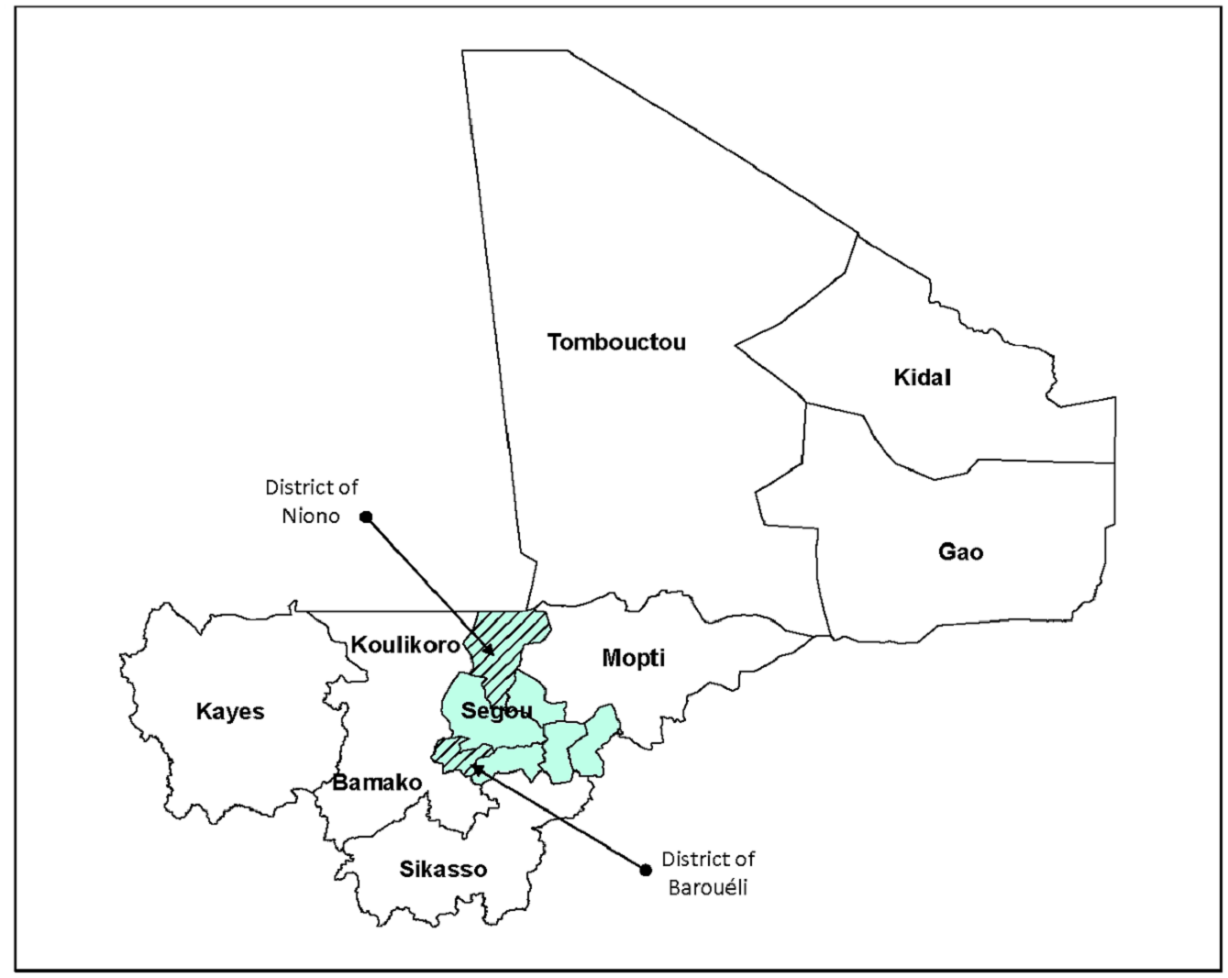
Map of Mali showing the two health districts selected for the RMM study in Ségou Region.

The community-based RMM study in Mali was conducted in the health districts of Niono and Barouéli, in the region of Ségou, located 240 km northeast of the capital, Bamako (Fig 1). The region of Ségou is mainly rural, with higher mortality and approximately the same level of fertility as the country as a whole [3].

The region of Ségou and the districts of Baraouéli and Niono were chosen in collaboration with the National Directorate of Health (DNS), the Regional Directorate of Health (DRS), and their partners. Initial selection criteria for the region and districts included the presence of UNICEF and the implementation of community case management (CCM) of childhood illness to permit the RMM results to contribute to an evaluation of the CCM program, although this evaluation did not materialize. Additional selection criteria were accessibility during the rainy season, and the judgment of the DNS and DRS with regards to whether the region and districts had functional management teams and networks of relais in place.

In each health district chosen for the study, we established a list of health facility catchment areas (19 for Niono, 23 for Barouéli). Two facility catchment areas were selected in each district (Molodo and Nara IBT in Niono; Kalakè and Sanando in Barouéli). After excluding catchment areas with populations of less than 5,000, we selected the RMM catchment areas based on the district health team’s assessment of whether the health facility was functional, the level of engagement by community organizations, and the existence of a functioning network of community relais.

The four selected facility catchment areas contained 49 villages (25 in Niono, 24 in Barouéli). The villages in each catchment area were then stratified according to the distance between the village and the first level health facility in the catchment area (<3 km, or ≥3 km). Within each stratum, one to five villages were sampled by systematic random sampling with probability proportional to the size of the village. In total, 20 villages were selected (S1 Table).

In the villages selected for the study, all of the households were eligible to participate, for a total of approximately 5,300 households. A household was defined as the head of household, all of his wives and their children residing in the compound, and the relatives living with them. Female-headed households were defined as a woman and her children residing in the compound, and any relatives living with them.

### Formative research

We conducted a cross-sectional qualitative assessment in May, 2010 in Niono and Barouéli districts to explore the current practices for recording vital events, actors involved in the recording of vital events, and barriers and local attitudes towards recording vital events data. The methods and results of this research are described in a separate manuscript [5].

### Implementation of community-based vital events reporting

#### Selection of cadre of community-based workers

Based on the results of the formative research, the *relais communautaires* were selected as the best-placed cadre to collect vital events data at the community level. Relais are an existing cadre of lay volunteer community workers in Mali who are responsible for health sensitization activities, as well as recording births and deaths in the community. Some relais have other leadership roles in the community, including teachers and traditional birth attendants. Given these roles, relais were identified by community members and health workers as a cadre that was both well-informed about pregnancies, births, and deaths in the village, and authorized to report these events.

All 78 relais in the 20 RMM villages participated in this study. The characteristics of these relais are provided in Table 1. No new relais were selected for the study, however, three relais died during the course of the study and were replaced by their communities. Study personnel played no role in choosing the replacements for these relais.

**Table 1:** Characteristics of relais participating in the RMM study in Mali.

	n (N = 78)	%
**Sex**		
Male	56	71.8%
Female	22	28.2%
**Age**		
20–29	6	7.7%
30–39	18	23.1%
40–49	29	37.2%
50+	17	21.8%
Not available	8	10.3%
**Highest level of education**		
None	2	2.5%
Literacy training in Bambara	48	61.5%
Koranic school/Madrassa	2	2.5%
Literacy training in French	4	5.1%
Primary school	18	23.1%
Secondary school	2	2.5%
Not available	2	2.5%

#### Training

Relais training was conducted from May to June, 2012. Seventy-eight relais were trained for three days at the referral health facility in each district. Training was conducted by a team of six trainers, including a study investigator, a field coordinator, a representative from the regional health directorate, a representative from the district health office, and two medical officers from first level facilities in the RMM area. The training focused on how to complete the pregnancy, birth, and death registers, and the importance of ensuring the accuracy and completeness of this information.

#### Community sensitization

In preparation for the RMM study, study investigators conducted sensitization activities with local authorities and local communities. In March, 2012, newsletters were sent to administrative and socio-health authorities (Regional Directorates of Health, Social Development, and Statistics) for the region of Ségou, the medical officers for the health districts of Niono and Barouéli, and the four technical directors of the health centers (Directeur Technique du Centre, or DTC) in the health catchment areas of the study. Study staff also visited the two districts to meet with officials from the Regional Directorate of Health and Social Development, Regional Directorate of Planning, Statistics, Information Technology and Land Use, the association of community health workers, and the chief medical officers and prefects of the two districts involved, to inform them about the RMM study.

At the community level, in June, 2012 study staff and local health officers held half day meetings in each RMM village to explain the study objectives and procedures, and the rationale for and importance of collecting information on vital events. These meetings were attended by village leaders, relais, and the leaders of women’s groups.

#### Data collection

Relais collected data on pregnancies, births, and deaths in the 20 RMM communities from June, 2012 to November, 2013. The pregnancy register also collected information on pregnancy outcomes, which were classified as live births, miscarriages and stillbirths. The month of June 2012 was a pilot period, and data from that month were discarded. Each relais was given three carbon paper registries—one each for births, deaths, and pregnancies—in either French or Bamanan (the local language) to record vital events (S1 Text). All but two study relais read and wrote French and/or Bamanan to some extent; the one relais who could not read or write was paired with another relais who helped him complete his registries.

Although relais are normally volunteers in Mali, we paid them 15,000 FCFA (approximately 30 USD) every three months, gave them phone cards to allow them to call their supervisor, and reimbursed them for travel to quarterly meetings.

#### Supervision and data quality assurance

The RMM project reinforced the supervision model of the Mali health system. RMM district coordinators established a field visit calendar with all RMM relais and with the community health worker (agent de santé communautaire or ASC) charged by the health system with supervising that relais. Field coordinators (hired for this study) supervised the relais every 15 days during the first two months of data collection, and monthly thereafter, often accompanied by ASCs.

The RMM research coordinator, RMM district coordinators, and representatives of the local health system supervised RMM relais quarterly. These supervision visits included a meeting in each district with all RMM relais in that district, in order to share experiences, examine registers to assess data quality, and address any problems. The visits also offered the opportunity to sensitize the relais on data quality issues and the importance of recording all events. Finally, study staff, medical officers from first-level and referral facilities, and representatives from the regional health directorate organized bi-annual supervision visits.

The RMM team put in place a number of procedures to ensure data quality, including verification of a 10% sample of reported births and deaths in randomly selected RMM villages during quarterly supervision visits, confirmation of the classification of all reported stillbirths with the health worker or traditional birth attendant who assisted the delivery or with the mother, and the provision of a list of pregnant women with estimated delivery dates in the next month (taken from the pregnancy register) to each relais on a monthly basis. The lists of women with an upcoming due date were shared with the relais starting in December, 2012, and were aimed at helping relais to monitor pregnancy outcomes and improve completeness of recording for births and early neonatal deaths.

#### Data management

The carbon copy sheets from the relais’ registers were double-entered each month. The resulting databases were compared and reconciled, and logic checks were conducted. Key variables were tabulated and the tables used to identify potential data quality issues monthly so that these could be addressed promptly. The results of these tabulations were fed back to relais during the quarterly supervision visits, and any data quality concerns were addressed with them.

### Validation of community-based vital events reporting

#### Design

We assessed the accuracy and completeness of the relais-reported data on births and under-five deaths by comparing these data to the results of a full pregnancy history for two rolling 12-month periods (July, 2012 to June, 2013 and October, 2012 to September, 2013). The full pregnancy history (hereafter referred to as the “validation survey”) was administered to women aged 15 to 49 years in a census of the households in RMM communities in November and December, 2013 (survey questionnaire available in S2 Text).

#### Training

Fifty-five interviewers and team leaders with at least a high school diploma, who were minimally computer literate and who spoke the local language (Bamanan) were selected for an eight-day training workshop. The workshop included training on study procedures, paper and electronic questionnaires in French and Bamanan, and human subjects research protections. In addition, interviewers and supervisors had two days of field practice in villages near Bamako. Team leaders received two days of additional training on supervision and data management procedures. Training was conducted by study investigators and staff, including the principal investigator. At the conclusion of the training, forty interviewers and eight team leaders were selected for the validation survey based on their performance and language skills.

#### Data collection

Data were collected by eight teams comprised of five interviewers and one supervisor. Data collection teams attempted to interview all households in the RMM communities. The study team had enumerated households in June, 2012, and in November and December, 2012, the list of households was updated and the compounds were mapped. For the validation survey, the household lists and maps from 2012 were used to locate households, and the relais in each village guided data collection teams to any compounds or households that had been established since December, 2012. Interviewers made up to three attempts to interview each household.

In each household, interviewers first listed the household members along with their age and sex in order to identify women aged 15 to 49 years who were eligible for the full pregnancy history (FPH) questionnaire. After providing consent, these women were asked about all of the pregnancies that they had had in their lifetime, including: the outcome of the pregnancy (live birth, stillbirth, or miscarriage); whether it was a multiple birth; sex, month and year of birth, and vital status of the child (for live births); and age at death (for live births that had died). Up to three attempts were made to interview each eligible woman.

#### Supervision

Data collection teams were supervised by two staff members from the National Statistics Institute in Ségou (DRPSIAP-Ségou) as well as the study data manager, all of whom had participated in the interviewer training and were present in the field for the entire survey period. In addition, study investigators conducted two, two-week supervision visits during data collection.

#### Data management

Data were collected on netbooks using CSPro software [6], and were downloaded and checked by team leaders each night. Data were compiled and checked by supervisors and central study staff weekly. Data were cleaned in CSPro and Stata, version 13 [7].

### Ethical review

Ethical clearance for the RMM study was obtained from the Johns Hopkins School of Public Health’s Institutional Review Board (IRB 3909) and the Ethical Review Committee, Faculty of Medicine, University of Bamako. Oral consent was obtained from study participants. We obtained a waiver of written consent from the IRB because much of the study population is illiterate. Oral consent was documented by study interviewers, who signed and dated the consent form after a study respondent provided their consent to participate. This procedure was approved by the Johns Hopkins School of Public Health IRB and the Ethical Review Committee, Faculty of Medicine, University of Bamako.

The anonymized datasets for this study are available at: DOI 10.7281/T1F769G3

### Analysis

Data on births and under-five deaths reported by relais in the period of July, 2012 to September, 2013 were included in the validation analysis. The data were analyzed for two rolling 12-month periods: July, 2012 to June, 2013, and October, 2012 to September, 2013. Data analysis was conducted in R version 3.0.2 (2013-09-25) [8].

We calculated the number of births and neonatal, infant, and under-five deaths reported by relais for each 12-month period. Because all relais submitted their vital event reporting forms for all 15 months of the RMM implementation period, we did not need to adjust the relais data for missing reports. We calculated neonatal, infant and under-five mortality rates for each period by dividing the number of these deaths documented by relais in a given period by the total births documented by relais in that same period.

The validation analysis involved two components: (1) an evaluation of the completeness of births and deaths reporting by relais; and (2) a comparison of under-five, infant, and neonatal mortality rates calculated from the relais data with those estimated from the validation survey for each 12-month period.

To evaluate the completeness of births and under-five deaths documented by the relais, we estimated the expected number of births and under-five deaths that should have been collected by relais for each 12-month period. We estimated the crude birth rate and the under-five mortality rate in each period directly from the validation survey. To estimate the expected number of births that should have been reported by relais, we multiplied the crude birth rate for each 12-month period by the total population size of the RMM catchment area in each district. The expected number of under-five deaths was calculated similarly, by multiplying the under-five mortality rate estimated from the validation survey by the expected number of births (calculated as described above). We examined the completeness of relais-based births and under-five deaths data by calculating the ratio of the total numbers of births and under-five deaths documented by relais to the expected numbers estimated as described above.

To compare under-five, infant, and neonatal mortality rates calculated from the relais data with those estimated from the validation survey, we calculated mortality rates from the relais data and the validation survey data by dividing the reported number of under-five deaths that occurred in each 12 month validation period by the number of live births that occurred during the same period. The mortality calculation was done in the same way for each data source to ensure direct comparability. Standard errors and confidence intervals were estimated using the jackknife resampling technique [9]. The Delta method [10] was used to examine the equivalence of the mortality rates based on the relais data with the rates from the validation survey. Statistical equivalence of the community-based and survey-based methods was assessed by calculating the ratio of the relais-based mortality rate to the validation survey-based mortality rate and the corresponding 95% confidence interval (CI), with a ratio of 1.0 indicating perfect statistical equivalence. If the upper bound of the 95% CI was less than 0.80, or the lower bound was greater than 1.20, we rejected the hypothesis of equivalence between the mortality rates produced by the two methods. These rejection criteria were defined a priori.

## Results

### Community-based vital events data

**Fig 2:**
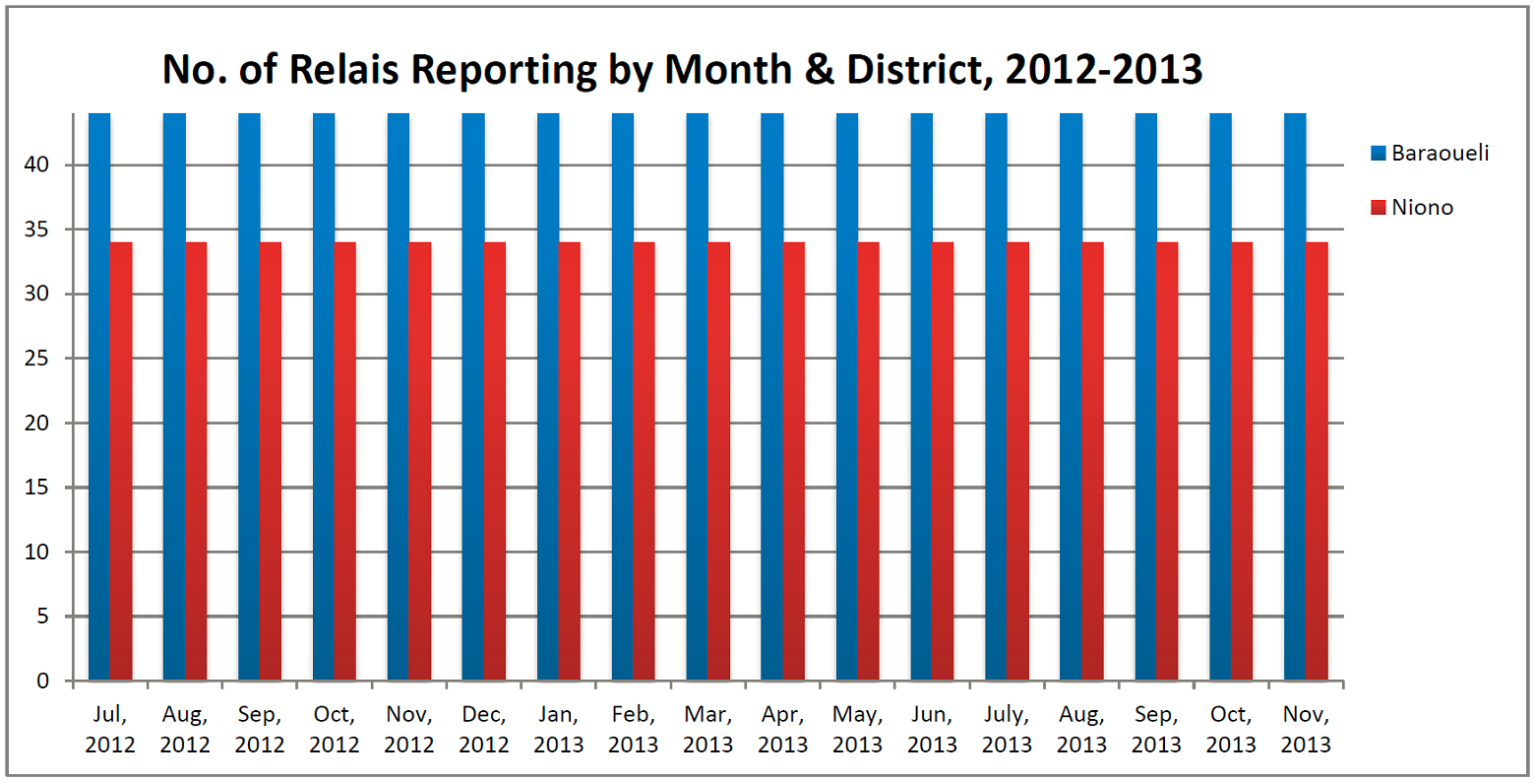
Number of relais catchment areas reporting data, by district.

In the two RMM districts, 78 relais in 20 villages conducted vital event reporting for the RMM project. These relais covered a total population of approximately 32,128 individuals, or 5,355 households. Monthly data on pregnancies, births, and deaths were consistently reported from 100% of the relais catchment areas (Fig 2).

In total, the relais reported 1,660 live births in the 17 months of RMM data collection—785 in Barouéli and 875 in Niono—and 37 stillbirths. The overall sex ratios at birth were 111 male births for every 100 female births in Barouéli and 128 in Niono. Relais reported a total of 276 deaths in the 17 months of data collection—127 in Barouéli and 149 in Niono. The age distribution of neonatal deaths and children who died between the ages of one and twenty-three months is reported in S1 and S2 Figs.

### Validation survey data

All 5,413 households in the RMM relais catchment areas were eligible to participate in the validation survey, and a total of 5,064 households (93.6%) were successfully interviewed. These interviews identified 6,533 eligible women (women aged 15–49 years), of whom 6,304 (96.5%) were interviewed. The main reason for household and women non-response was households or individuals who were absent; interviewers reported that most absences were due to the harvest or other work, as the survey was conducted during the harvest.

Interviewed women reported 26,165 pregnancies over the course of their lives, of which 904 (3.5%) ended in a stillbirth, 1,575 (6%) in a miscarriage, and 23,686 in a live birth. The average sex ratio in the validation survey was 109 male births for every 100 female births in Barouéli, and 114 in Niono. The age distribution of neonatal deaths and children who died between the ages of one and twenty-three months in the validation survey is reported in S3 and S4 Figs.

### Validation

Table 2 presents the total number of births and under-five deaths reported by relais compared to the number of births and under-five deaths expected to be reported based on our validation survey estimates and the population. For both births and deaths, the relais documented approximately 90% of the number of events expected based on the validation survey estimates.

**Table 2:** Births and under-five deaths reported by relais compared to expected births and deaths based on validation survey.

Annual periods	Estimated total population of RMM area	Estimated crude birth rate (Validation survey, per 1,000)	U5MR (Validation survey, per 1,000)	Births			Under Five Deaths		
				Expected number of births	No. births reported by relais	Ratio of births reported by relais to expected births (%)	Expected number of U5 deaths	Number of U5 deaths reported by relais	Ratio of U5 deaths reported by relais to expected U5 deaths (%)
**Barouéli**									
Jul 2012–Jun 2013	15092	43.1	131.7	650	579	89.1	86	77	90.0
Oct 2012–Sep 2013	15092	44.8	130.1	677	585	86.5	88	78	88.6
**Niono**									
Jul 2012–Jun 2013	17037	39.3	177.6	670	609	90.9	119	109	91.6
Oct 2012–Sep 2013	17037	38.5	153.4	656	617	94.1	101	94	93.5
**Total**									
Jul 2012–Jun 2013	32129	41.0	155.9	1318	1188	90.1	206	186	90.5
Oct 2012–Sep 2013	32129	41.4	142.0	1329	1202	90.4	189	172	91.1

Table 3 compares the under-five, infant, and neonatal mortality rates estimated from the relais data to those estimated in the validation survey. The under-five mortality rate calculated from the relais data was practically and statistically equivalent to that estimated using the validation survey. The under-five mortality rates from the community-based method and survey-based method differed by one death per 1000 or less, and the ratios of the community-based to the survey-based mortality rates were 1.00 (95% CI: 0.80, 1.201) and 1.01 (95% CI: 0.80, 1.22) for the reporting periods, indicating statistical equivalence of the methods. However, the relais-reported infant mortality rate (IMR) and neonatal mortality rate (NMR) were greater than the IMR and NMR estimated from the validation survey.

**Table 3:** Neonatal, infant, and under-five mortality rates from relais records and validation survey and associated confidence intervals.

Annual Periods	Neonatal Mortality Rate (per 1,000)						Infant Mortality Rate (per 1,000)						Under-Five Mortality Rate (per 1,000)					
	Relais data		Validation Survey		Ratio of Mortality Rates, Relais Data to Validation Survey		Relais data		Validation Survey		Ratio of Mortality Rates, Relais Data to Validation Survey		Relais data		Validation Survey		Ratio of Mortality Rates, Relais Data to Validation Survey	
	Rate	95% CI	Rate	95% CI	Ratio	95% CI	Rate	95% CI	Rate	95% CI	Ratio	95% CI	Rate	95% CI	Rate	95% CI	Ratio	95% CI
**Barouéli**																		
Jul 2012–Jun 2013	36.3	(20.8, 51.8)	27.7	(16.1, 39.3)	1.31	(1.15, 1.47)	67.4	(46.2, 88.5)	71.1	(50.5, 91.5)	0.95	(0.84, 1.05)	133.0	(105.3, 160.6)	131.7	(105.7, 157.7)	1.01	(0.78, 1.24)
Oct 2012–Sep 2013	35.9	(20.5, 51.3)	30.4	(20.0, 40.8)	1.18	(1.03, 1.34)	85.5	(61.8, 109.2)	67.6	(48.6, 86.5)	1.27	(1.16, 1.37)	133.3	(105.7, 160.9)	130.1	(104.8, 155.5)	1.03	(0.80, 1.25)
**Niono**																		
Jul 2012–Jun 2013	49.3	(31.6, 66.9)	40.5	(29.3, 51.7)	1.22	(1.08, 1.35)	101.8	(76.5, 127.1)	88.8	(67.3, 110.3)	1.15	(1.06, 1.24)	179.0	(148.5, 209.4)	177.6	(148.7, 206.5)	1.01	(0.82, 1.20)
Oct 2012–Sep 2013	53.5	(35.2, 71.7)	36.7	(24.9, 48.5)	1.46	(1.32, 1.60)	97.2	(72.6, 121.9)	68.7	(49.3, 88.1)	1.42	(1.31, 1.52)	152.4	(124.0, 180.7)	153.4	(125.8, 180.9)	0.99	(0.78, 1.21)
**Total**																		
Jul 2012–Jun 2013	42.9	(31.1, 54.7)	34.5	(18.5, 38.7)	1.24	(1.11, 1.38)	85.0	(68.4, 101.6)	80.4	(65.7, 95.1)	1.06	(0.97, 1.15)	156.6	(135.9, 177.2)	155.9	(136.3, 175.5)	1.00	(0.80, 1.21)
Oct 2012–Sep 2013	44.9	(32.9, 56.9)	33.7	(17.9, 39.5)	1.33	(1.20, 1.47)	91.5	(74.4, 108.6)	68.1	(54.6, 81.7)	1.34	(1.25, 1.44)	143.1	(123.2, 162.9)	142.0	(123.2, 160.8)	1.01	(0.80, 1.22)

## Discussion

### Summary and interpretation of key findings

This study sought to assess the ability of community-based workers in Mali to record vital events data (births and under-five deaths) accurately and completely. The study strengthened the supports available to the relais by: hiring two full-time field coordinators (one for every 10 villages) who conducted monthly supervision, data verification, and data entry activities; analyzing data on an ongoing basis and providing feedback to relais at quarterly meetings; and providing compensation to relais in the form of quarterly stipends and airtime. In addition, the study included strong community sensitization regarding the importance of vital events reporting, and was conducted in a relatively small population over a relatively short time period. The results of this study therefore provide information about the ability of a community-based system to provide accurate mortality information in a small population given relatively strong supports. This study was not a test of the scalability of this method and does not provide an indication of how such a method would perform at scale.

We found that both the community-based method and the validation survey produced relatively high quality data. The sex ratios at birth, however, which are expected to fall between 102 and 107 male births per 100 female births in most populations [11], suggested some underreporting of female births relative to male births, particularly in Niono district, for the community-based data and the validation data. We also saw indications of age heaping, where respondents round the reported age to the nearest 6 or 12 months, for age at death in the validation study, especially at 12 months of age. This finding is not surprising for a retrospective mortality survey, where the mother may not be able to recall the child’s exact age at death with precision. Notwithstanding these issues, we believe that the quality of the data is sufficiently high to allow for a valid test of the performance of the community-based method in estimating mortality over 12-month periods.

The community-based method in Mali produced estimates of the under-five mortality rate that were statistically and practically equivalent to those produced by the full pregnancy history in the validation survey. By practical equivalence we mean that the estimates produced by both methods were very close (a difference of one death per 1000 live births) and therefore interchangeable for the purposes of tracking progress in reducing under-five mortality. At small scale, this method therefore performed as well as a standard retrospective mortality survey with full pregnancy history in producing estimates of under-five mortality. This finding suggests that, with relatively strong supervision and incentives, community-based workers are capable of collecting data that would allow governments and programs to track changes in under-five mortality on an annual basis. It is not clear, however, whether the quality of the data collected by these workers could be maintained if the program were scaled up, nor whether such a large-scale program would be cost effective.

Although surveys with full birth histories or full pregnancy histories are generally considered to be the best available approach for producing estimates of mortality rates in developing countries, we found that the community-based method produced higher estimates of early mortality, particularly neonatal mortality, relative to the retrospective validation survey. As it is unlikely that women would report deaths that did not occur, these higher rates could be due to lower reporting of births in the community-based method than in the validation survey, higher reporting of early deaths in the community-based method than the validation survey, or misclassification of stillbirths or early neonatal deaths in the community-based method or validation survey. Births do not seem to have been under-reported by the community-based method in comparison to the validation survey. A comparison of the patterns of neonatal deaths by age at death (in days) in the community-based and survey-based methods, relative to the expected daily risk of death [12] reveals some misclassification of day zero deaths as day one deaths in the validation survey, and a slightly higher than expected proportion of neonatal deaths in the first day of life for the community-based method in the second 12-month validation period (S5 and S6 Figs). This could indicate misclassification of some stillbirths as early neonatal deaths in the second validation period, which would account in part for the higher neonatal mortality rate observed with the community-based method.

There are plausible explanations for the increased reporting of early deaths in the community-based method in Mali. This method was prospective and included reporting pregnancies, which could then be followed to obtain pregnancy outcomes. District coordinators and central study staff emphasized to relais the importance of having an outcome for each pregnancy, and the importance of reporting all deaths, even those that occurred very early. Finally, the community-based method used relais, who are well-known members of the community, to report births and deaths. Their status as respected community members who conduct some health sensitization activities may have given them an advantage in learning about births and deaths, relative to the interviewers used in the validation survey, who were not from the community.

There is some qualitative evidence suggesting that in developing countries, perinatal events (stillbirths and early neonatal deaths) are highly sensitive and are not publicly discussed or disclosed [13]. Further, there may be confusion as to the classification of early events; the medical terms “miscarriage,” “stillbirth,” and “neonatal death” do not always have equivalents in local languages, and perinatal losses may be categorized more in terms of the baby’s size or appearance (large, “mature”, or “immature”) than whether the event happened before, during, or after birth [13]. A few studies have attempted to assess the validity of retrospective survey-based data on neonatal and child deaths, either through an internal data quality assessment or by comparison with demographic surveillance-type data. The results have been mixed, with several studies showing slightly higher infant mortality estimates from surveillance compared to retrospective surveys, but no difference for neonatal deaths [14, 15, 16]. Haws et al. also cited unpublished findings from Uttar Pradesh, India, showing a much higher neonatal mortality rate measured through prospective surveillance than through a retrospective mortality survey [13].

### Limitations

Our study has several limitations. Although ideally we would have validated the community-based method against a method that recorded all births and deaths, no such method exists. Although surveys with retrospective pregnancy histories are considered the best available method for collecting data, particularly in large populations, they do not capture all births and deaths due to recall error, misreporting, and interviewer error. Thus, our validation of the community-based method was closer to an equivalence test against the current standard for collecting mortality data for children under five years.

Second, the community-based method was implemented over a relatively short period of time (17 months). It is possible that when implemented over a longer period of time, the completeness of reporting would deteriorate. Routine quality control and continuous field supervision may be difficult to sustain over longer periods of time.

Third, our validation analysis assumes that migration in and out of the study area is negligible during the 17-month reference period. One of the villages in the RMM area had a large population of Fulani (a nomadic ethnic group), and relais in this village reported difficulty in tracking births and deaths, which were often reported months after the fact if they occurred while a household was away from the village. This village represented approximately 6% of the population of the RMM area, and the other villages within the study area had very little migration. Hence, community-based vital events reporting might not work as well for areas with non-negligible levels of migration. For urban areas and for the northern regions of Mali, which have large nomadic populations and have been experiencing considerable conflict-related migration, there may be inconsistencies between the vital events that are observable by relais and those that can be reported retrospectively by a sample of households.

Fourth, this study did not involve systematic active surveillance of pregnancies. Rather, relais reported pregnancies when women shared that information with community members and reported on the outcome when women informed them of the birth, stillbirth, or miscarriage. We encouraged relais to follow up with women with approaching due dates to learn about the outcome of the pregnancy, but this was not done in a systematic way, which may have resulted in underreporting of pregnancy losses. A potential refinement of the RMM community-based method might involve more proactive and detailed pregnancy status tracking so as to reduce potential misclassification errors, although this would likely require increased supervision of relais.

Finally, the validation analysis we present in this paper evaluates the consistency of annualized birth counts, death counts, and under-five mortality rates between two data collection systems as opposed to assessing the consistency of record-level reporting of vital events information between the two systems. In a separate analysis within this collection, Silva et al. report on a record-linkage study that evaluates the consistency of reporting between both data collection methods and the level of under-reporting of birth events and death events by both systems.

## Conclusions

This assessment suggests that community-based methods for vital events reporting built on the existing system for reporting these events holds promise for obtaining data on mortality at 12-month intervals on a small scale, and potentially on a larger scale, if adequate supports are put into place. The community-based method tested in Mali performed well when implemented in 20 villages with strong supports, and should be considered for gradual scale-up in Mali, with appropriate measures to ensure community and relais engagement, close supervision of relais, ongoing data analysis and data quality assessment, and cross-validation with comparable FPHs.

## Supporting Information

S1 Fig Distribution of neonatal deaths by age at death in days, Mali community-based data, July 2012-November 2013. (PDF)

S2 Fig Distribution of deaths from 1 to 23 months by age at death in months, Mali community-based data, July 2012-November 2013. (PDF)

S3 Fig Distribution of neonatal deaths by age at death in days, Mali RMM validation survey, November-December 2013. (PDF)

S4 Fig Distribution of deaths from 1 to 23 months by age at death in months, Mali RMM validation survey, November-December 2013. (PDF)

S5 Fig Cumulative percentage of neonatal deaths by day of death, July 2012-June 2013. (PDF)

S6 Fig Cumulative percentage of neonatal deaths by day of death, October 2012-September 2013. (PDF)

S1 Table List of RMM villages. (DOCX)

S1 Text Relais vital event reporting forms. (PDF)

S2 Text Validation survey questionnaire. (PDF)
