# Supplementary figures and images for: Strengthening Community Networks for Vital Event Reporting: Community-Based Reporting of Vital Events in Rural Mali
Source: PLoS One. 2015 Nov 25;10(11):e0132164. doi: 10.1371/journal.pone.0132164 (PMC4659620; doi:10.1371/journal.pone.0132164)

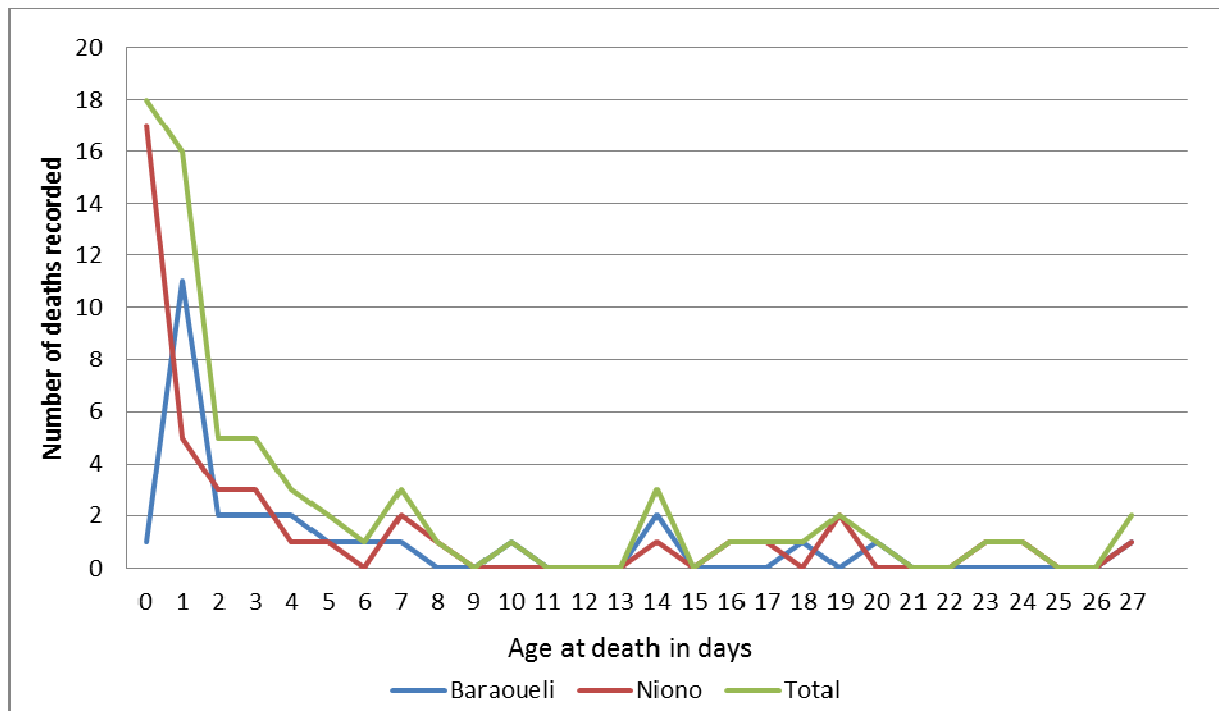

Supplement: S1 Fig — (PDF) [file pone.0132164.s001.pdf]

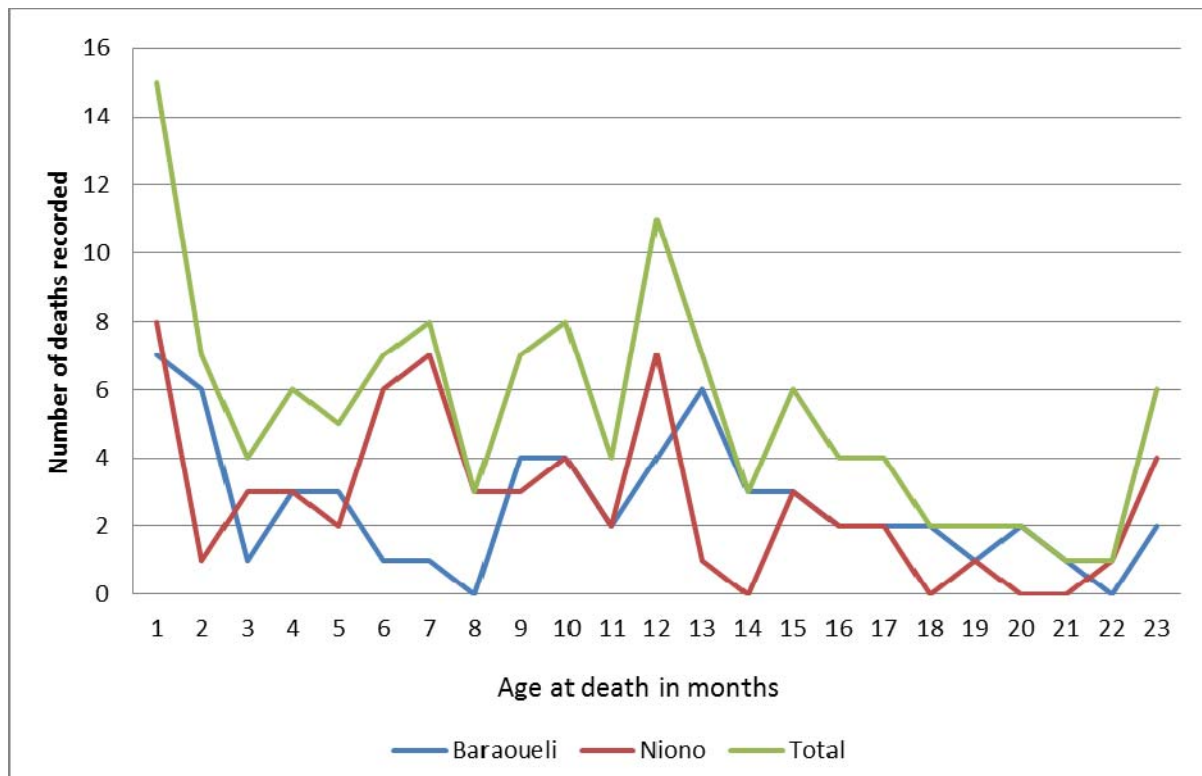

Supplement: S2 Fig — (PDF) [file pone.0132164.s002.pdf]

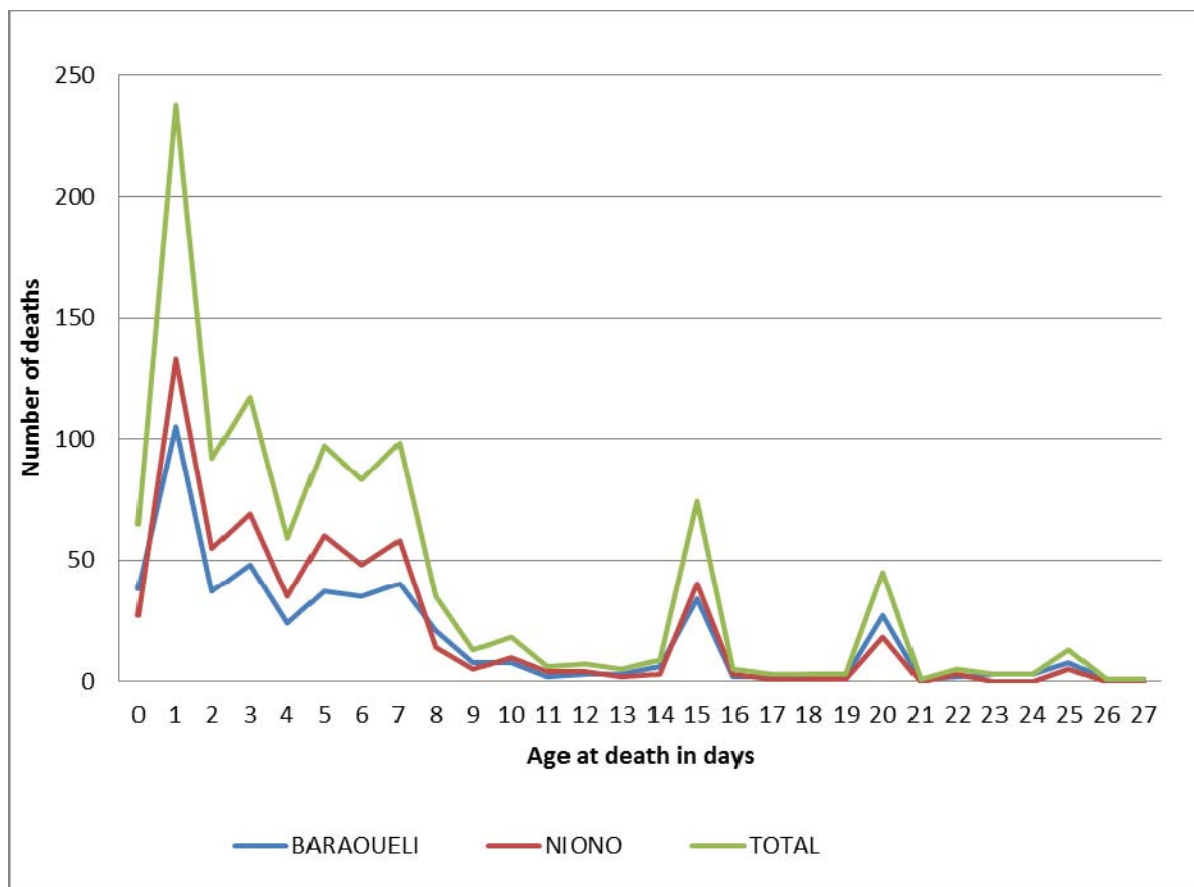

Supplement: S3 Fig — (PDF) [file pone.0132164.s003.pdf]

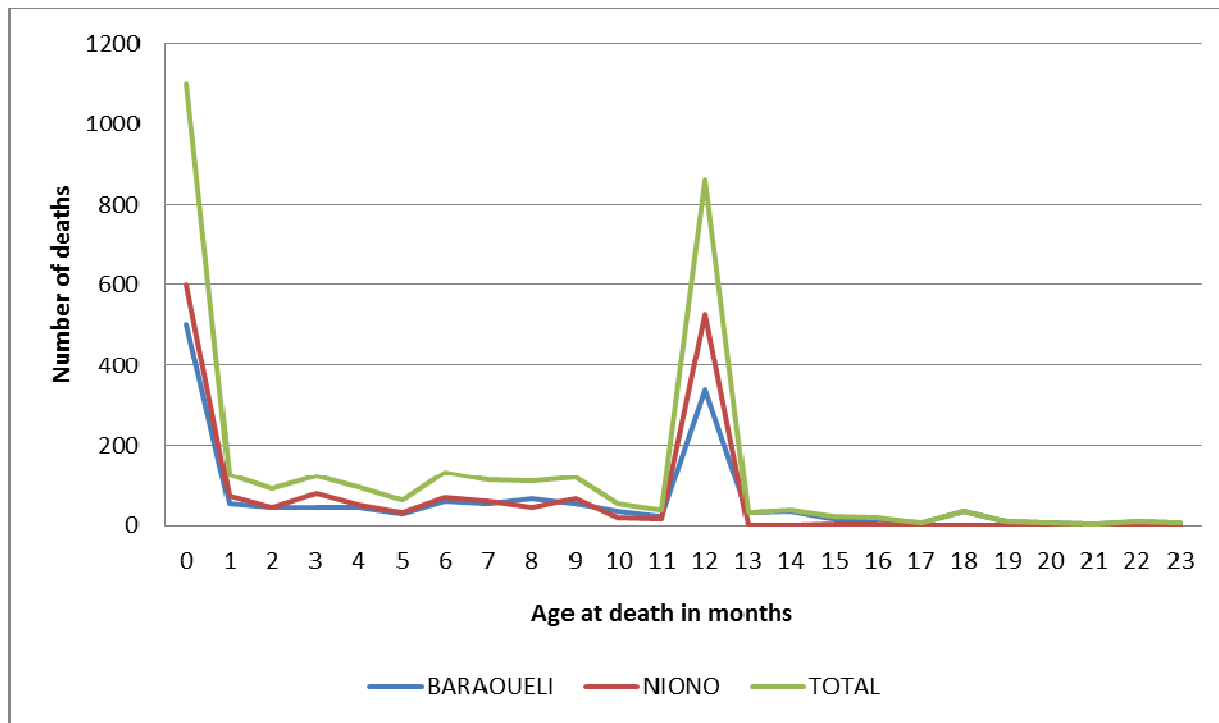

Supplement: S4 Fig — (PDF) [file pone.0132164.s004.pdf]

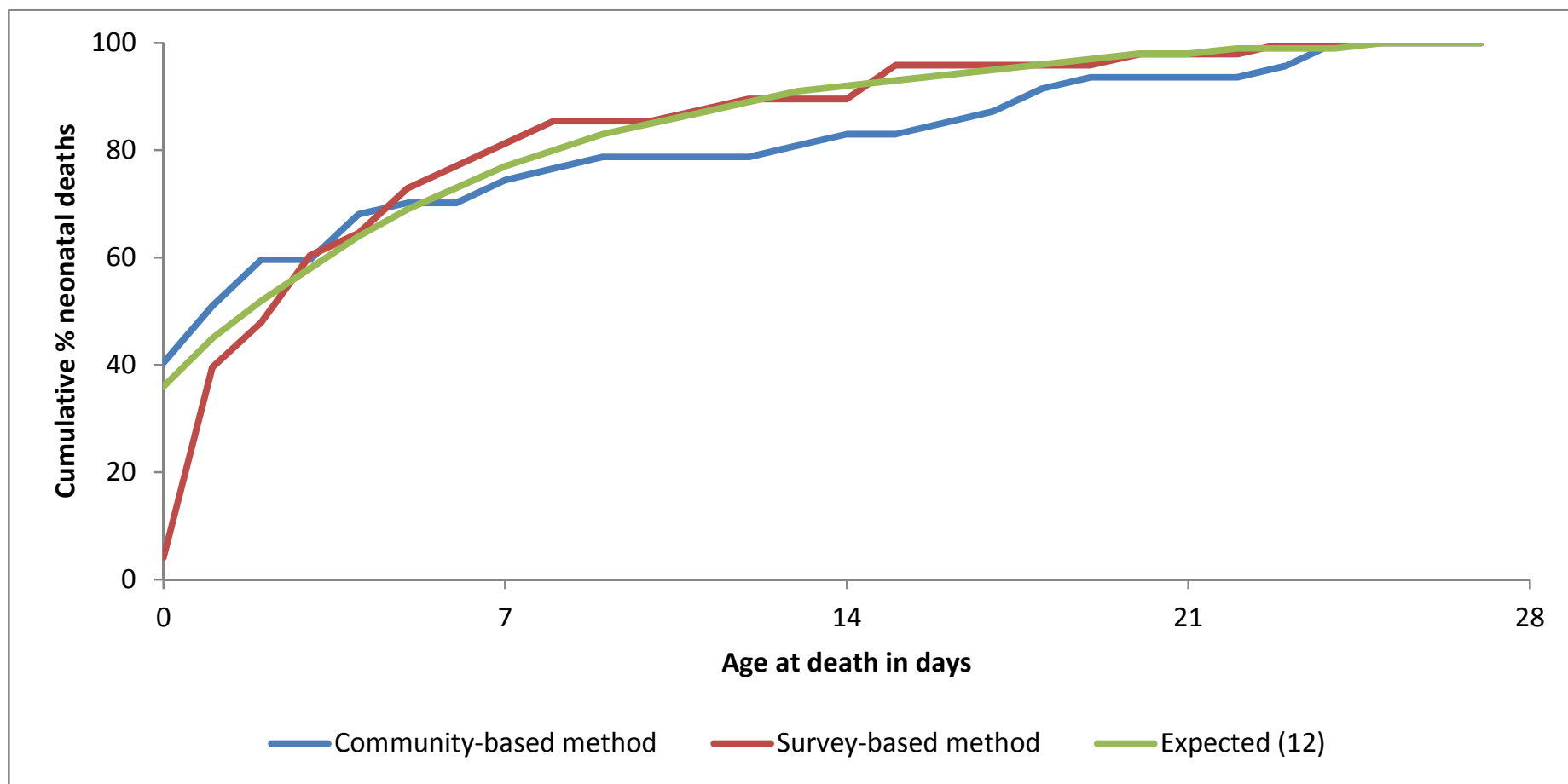

Supplement: S5 Fig — (PDF) [file pone.0132164.s005.pdf]

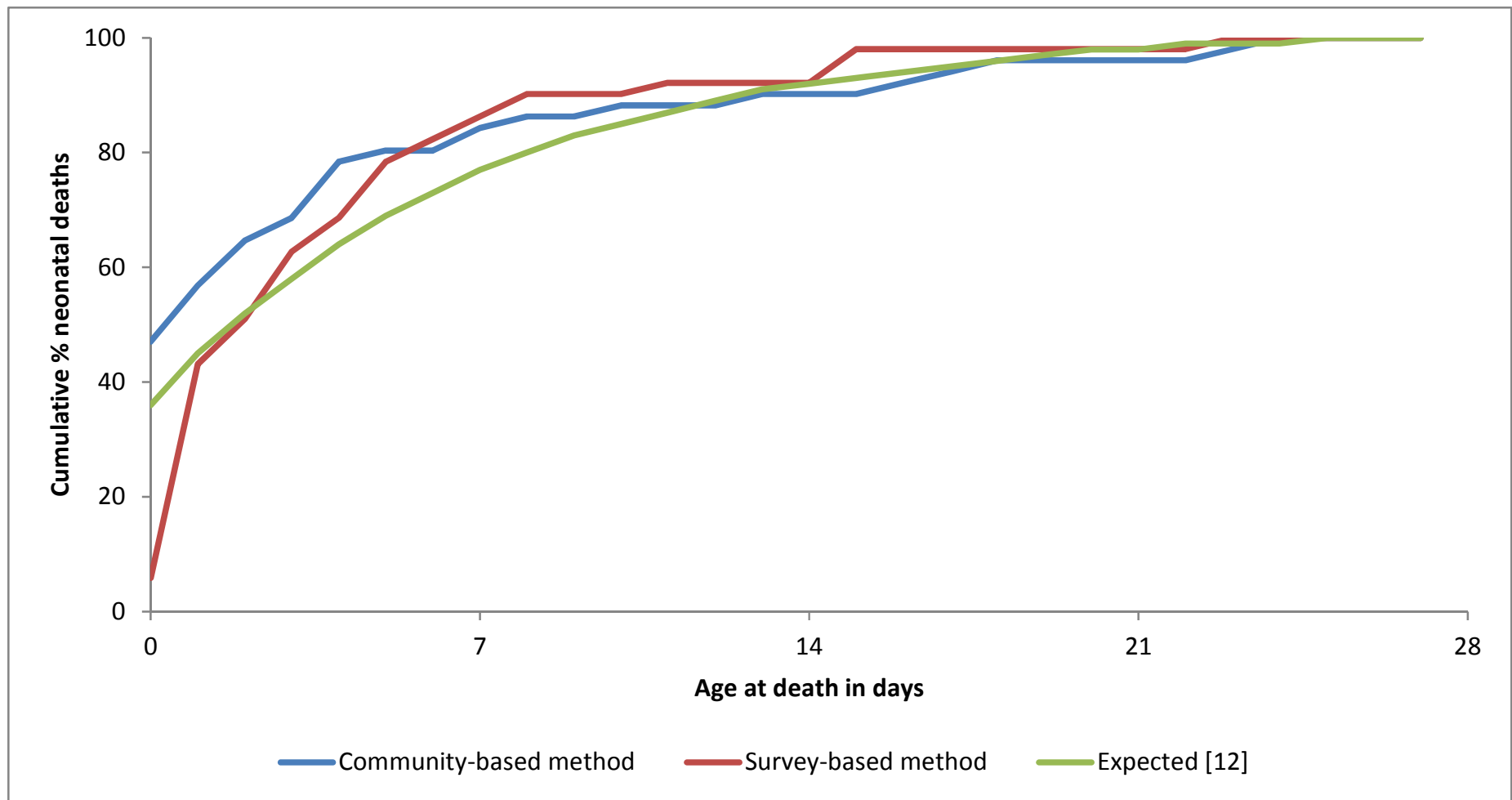

Supplement: S6 Fig — (PDF) [file pone.0132164.s006.pdf]
