# Supplementary material for: Strengthening Community Networks for Vital Event Reporting: Community-Based Reporting of Vital Events in Rural Mali
Source: PLoS One. 2015 Nov 25;10(11):e0132164. doi: 10.1371/journal.pone.0132164 (PMC4659620; doi:10.1371/journal.pone.0132164)
Supplement: S1 Table — (DOCX) [file pone.0132164.s007.docx]

**S1 Table. List of RMM villages**

| Villages | Distance (km) from village to 1st level facility | Estimated Population 2011 | Estimated number of households |
| --- | --- | --- | --- |
| **NIONO- MOLODO** | | | |
| MOLODO CENTRE | 0 | 4739 | 803 |
| NEMABOUGOU | 4 | 315 | 53 |
| MANIALE | 8 | 1974 | 335 |
| NIAFASSI MARKA | 8 | 765 | 130 |
| BAKAYE-WERE | 10 | 876 | 148 |
| **NIONO- NARA IBT** | | | |
| NARA | 0 | 1593 | 270 |
| TIGABOUGOU | 4 | 534 | 91 |
| N'GOUNANDO KORO | 7 | 826 | 140 |
| SIGUIWOUCE | 10 | 1224 | 208 |
| **BARAOUELI- KALAKE** | | | |
| KALAKE MARKA | 3 | 5116 | 493 |
| BERTELA | 3 | 1008 | 171 |
| DIAWARALA | 3 | 1689 | 115 |
| BEYA | 4 | 2920 | 94 |
| ZANFINA 1 | 8 | 4790 | 109 |
| **BARAOUELI- SANANDO** | | | |
| SANANDO | 0 | 1745 | 296 |
| KOYA | 3 | 1284 | 148 |
| TOMI | 5 | 3032 | 108 |
| WOLOKORO | 7 | 4307 | 120 |
| BOLITOMO | 7 | 6305 | 225 |
| KONI | 11 | 8163 | 143 |
