# Supplementary material for: Strengthening Community Networks for Vital Event Reporting: Community-Based Reporting of Vital Events in Rural Mali
Source: PLoS One. 2015 Nov 25;10(11):e0132164. doi: 10.1371/journal.pone.0132164 (PMC4659620; doi:10.1371/journal.pone.0132164)
Supplement: S1 Text — (PDF) [file pone.0132164.s008.pdf]

CREDOS

FORMULAIRE DE COLLECTE DE GROSSESSE

District : ____________ **Aire de santé :** ________________ **Village :** ______________ **Relais :** _____________________________

Collecte du mois de : ________________________________ **Nombre Total de Grossesses :** [ ]

N°	Date Enrg jj/mm/aa	GROSSESSES												Commentaires
		N° C	N° M	Nom Femme enceinte	Age	Nom du Conjoint	Age Grossesse En mois	Date PA jj/mm/aa	Issue de la Grossesse			Date Issue jj/mm/aa	
									Né vivant	Mort-né	Avorté		
1	__/__/__							__/__/__				__/__/__	
2	__/__/__							__/__/__				__/__/__	
3	__/__/__							__/__/__				__/__/__	
4	__/__/__							__/__/__				__/__/__	
5	__/__/__							__/__/__				__/__/__	
6	__/__/__							__/__/__				__/__/__	
7	__/__/__							__/__/__				__/__/__	
8	__/__/__							__/__/__				__/__/__	
9	__/__/__							__/__/__				__/__/__	
10	__/__/__							__/__/__				__/__/__	

Date Enrg = Date enregistrement des données ; **Date PA**= Date Probable d'Accouchement; **N°C** : Numéro concession ; **N° M** : Numéro Ménage

Première saisie : Par _______________________ **:** ***Date :*** __/__/__

Deuxième saisie : Par _______________________ **:** ***Date :*** __/__/__

FORMULAIRE DE COLLECTE DE NAISSANCE

District : ____________ **Aire de santé :** ________________ **Village :** ______________ **Relais :** ______________________________

Collecte du mois de : ________________________________ **Nombre Total de Naissances :** [ ]

N°	Date Enrg. jj/mm/aa	NAISSANCES						Lieu de Naissance			Sexe		Né Vivant		Commentaires
		No C.	No M.	Nom du Père	Nom de la Mère	Nom de l'enfant	Date Nais. jj/mm/aa	Domicile	Centre de santé	Autre	M	F	OUI	NON	
1	__/__/__						__/__/__								
2	__/__/__						__/__/__								
3	__/__/__						__/__/__								
4	__/__/__						__/__/__								
5	__/__/__						__/__/__								
6	__/__/__						__/__/__								
7	__/__/__						__/__/__								
8	__/__/__						__/__/__								
9	__/__/__						__/__/__								
10	__/__/__						__/__/__								

Date Enrg = Date enregistrement des données ; **Date Nais** = Date de Naissance ; **M**= Masculin ; **F**= Féminin ;**N°C** : Numéro concession ; **N° M** : Numéro Ménage

Première saisie : Par ______________________ ***:*** ***Date :*** __/__/__

Deuxième saisie : Par ______________________ ***:*** ***Date :*** __/__/__

FORMULAIRE DE COLLECTE DE DECES

District : ____________ **Aire de santé :** ________________ **Village :** ______________ **Relais :** ______________________________

Collecte du mois de : _________________________________ **Nombre Total de Décès :** [ ]

N°	Date Enrg. jj/mm/aa	N° C	N° M	DECES													Commentaires
				Nom du Père	Nom Mère	Nom Enfant	Date Naissance jj/mm/aa	Date Décès jj/mm/aa	Age au moment du Décès		Sexe		SP le décès	Lieu de décès			
									Jour	Mois	M	F		Domicile	Centre de santé	Autre	
1	__/__/__						__/__/__	__/__/__									
2	__/__/__						__/__/__	__/__/__									
3	__/__/__						__/__/__	__/__/__									
4	__/__/__						__/__/__	__/__/__									
5	__/__/__						__/__/__	__/__/__									
6	__/__/__						__/__/__	__/__/__									
7	__/__/__						__/__/__	__/__/__									
8	__/__/__						__/__/__	__/__/__									
9	__/__/__						__/__/__	__/__/__									
10	__/__/__						__/__/__	__/__/__									

Date Enrg = Date enregistrement des données ; **SP le décès** = Signes précédents le décès; **M**= Masculin ; **F**= Féminin ; **N°C** : Numéro concession ; **N° M** : Numéro Ménage

Première Saisie Par : ____ **Date :** ___/___/_____

Deuxième Saisie Par : ____ **Date :** ___/___/_____
