# Supplementary material for: Strengthening Community Networks for Vital Event Reporting: Community-Based Reporting of Vital Events in Rural Mali
Source: PLoS One. 2015 Nov 25;10(11):e0132164. doi: 10.1371/journal.pone.0132164 (PMC4659620; doi:10.1371/journal.pone.0132164)
Supplement: S2 Text — (PDF) [file pone.0132164.s009.pdf]

INFORMATION MENAGE — HH

HH1. DISTRICT<br>BARAOUELI .... 1<br>NIONO 2	HH2. AIRES DE SANTE<br>MOLODO .... 1<br>NARA IBT .... 2<br>KALAKE .... 3<br>SANANDO 4
HH3. Nom et Numéro du Village:<br>Non ____________ Numéro ______	HH4. Nom et Numéro du Relais<br>Nom: ____________ Numéro ______
HH4. Numéro de concession: R ___ ___ | C ___ ___	HH5. Numéro de ménage (de la base recensement)<br>___ ___ ___ ___
HH6. Nom et Numéro l'enquêteur (trice):<br>Non ____________ Numéro ______	HH7. Nom et Numéro du contrôleur<br>Nom: ____________ Numéro ______

HH8. Jour/ Mois ________/______/2013

Lire le formulaire de consentement Ménage au répondant du questionnaire Ménage avant de poursuivre l'interview.

La permission est-elle accordée?

☐ *Oui, la permission est accordée* → *Allez à HL2 (page suivante) pour enregistrer le temps et ensuite démarrer l'interview.*

☐ *Non, la permission n'est pas accordée* → *Complétez HH11. Discutez de ce résultat d'interview avec votre contrôleur*

Une fois que tout le questionnaire Ménage sera rempli, vous devriez completer les parties ci-dessous:

HH9. Nom du chef de Ménage: ______________________________

HH11. Résultat de l'enquête Ménage<br>Rempli .... 01<br>Pas de membre dans le ménage ou pas de membre éligible au moment de la visite .... 02<br>Tous les membres du ménage sont absents pour une longue durée .... 03<br>Réfusé .... 04<br>Ménage vacant / pas de ménage à l'adresse .... 05<br>Ménage détruit .... 06<br>Ménage non-trouvé .... 07<br>Autre (précisez): ____________ 96	HH12. Répondant du Questionnaire Ménage<br>Nom: ______________________<br>Numéro de ligne: ________<br>HH13. Nombre total de membres de Ménage: ________<br>HH14. Nombre de femmes âgées de 15-49 Ans: ________<br>HH15. Nombre de Questionnaires Femmes remplis: ________

Notes de l'enquêteur (trice)

HH16. Contrôleur (Nom et Numéro): ____________________ ________

HH17. Enregistrez l'heure.	Heures et minutes: ___ ___: ___ ___

FICHE DE DENOMBREMENT DES MENAGES (LISTE DES MEMBRES DU MENAGE) HL

S'il vous plait, donnez-moi le prénom puis le nom des personnes qui vivent habituellement dans votre ménage ainsi que les visiteurs qui ont passé la nuit dernière chez vous. Commencez par le chef de ménage sur la Ligne 01. *Listez tous les membres du ménage (HL2), leurs liens de parenté avec le chef de ménage (HL3) et leur sexe (HL4)*

Demandez ensuite : Y a-t-il d'autres membres qui vivent dans le ménage, même s'ils ne sont pas à la maison présentement? (Ceci pourrait inclure les personnes à l'école, au travail ou au champ).*Si oui, compléter la liste de questions HL2-HL4 Ensuite, poser le reste des questions, en commençant parHL5 pour une personne à la fois.*

											Pour les membres du ménage âgées de 15 ans et plus	Femme âgée de 15-49 ans				Pour tous les membres du ménage
HL1 N0. Ligne	HL2 PRENOM ET NOM	HL3.QUEL LIEN DE PARENTE ENTRE (PRENOM) ET LE CHEF DE MENAGE ?	HL4. DE QUEL SEXE (MASCULIN OU FEMININ) EST (NOM)?<br>1 Masculin<br>2 Féminin		HL5A. (PRENOM) RESIDE-T-IL HABITUELLEMENT DANS CE MENAGE?<br>1 Oui<br>2 Non		HL5. QUELLE EST LA DATE DE NAISSANCE DE (PRENOM)?<br>98 NSP 98 NSP 9998 NSP<br>S'assurer que toutes les personnes de moins de 6 ans ont le mois et l'année de naissance renseignés.			HL6. QUEL ÂGE A (PRENOM)?<br>Enregistrez en années révolues<br>Si 95 ans ou plus, enregistrez '95'	HL7. QUELLE EST ACTUELLEMENT LA SITUATION MATRIMONIALE DE (PRENOM) ?<br>1 Marié ou en couple<br>2 Divorcé / Séparé<br>3 Veuf (ve)<br>4 Célibataire	HL8. Entourez le numéro de ligne s'il s'agit d'une femme âgée de 15-49 ans	HL9. Numéro de Ménage (M) de la femme<br>Si 99, allez à HL10; sinon allez à HL9A	HL9A. Numéro de Ménage (M) de la mère de la femme<br>Si la femme ne dépend pas de sa mère, enregistrez 00	HL10. Numéro d'identifiant de la femme<br>Si la femme n'a pas de carte, enregistrez 9999999	HL11. DEPUIS COMBIEN D'ANNEES (PRENOM) VIT-IL/ELLE DANS CE VILLAGE ?<br>Enregistrez 0 si moins d'une année
Ligne	Prénom et Nom	Lien de parenté	M	F	O	N	jj	mm	Année	Age	Situation matrimoniale	15-49				Années
01		01	1	2	1	2	___ ___	___ ___	___________		1 2 3 4	1	___ ___	___ ___	__ __ __ __ __ __ __	
02			1	2	1	2	___ ___	___ ___	___________		1 2 3 4	2	___ ___	___ ___	__ __ __ __ __ __ __	
03			1	2	1	2	___ ___	___ ___	___________		1 2 3 4	3	___ ___	___ ___	__ __ __ __ __ __ __	
04			1	2	1	2	___ ___	___ ___	___________		1 2 3 4	4	___ ___	___ ___	__ __ __ __ __ __ __	
05			1	2	1	2	___ ___	___ ___	___________		1 2 3 4	5	___ ___	___ ___	__ __ __ __ __ __ __	
06			1	2	1	2	___ ___	___ ___	___________		1 2 3 4	6	___ ___	___ ___	__ __ __ __ __ __ __	
07			1	2	1	2	___ ___	___ ___	___________		1 2 3 4	7	___ ___	___ ___	__ __ __ __ __ __ __	
08			1	2	1	2	___ ___	___ ___	___________		1 2 3 4	8	___ ___	___ ___	__ __ __ __ __ __ __	

09			1	2	1	2	___ ___	___ ___	__________		1 2 3 4	9	___ ___	___ ___	_ _ _ _ _ _ _ _	
10			1	2	1	2	___ ___	___ ___	__________		1 2 3 4	10	___ ___	___ ___	_ _ _ _ _ _ _ _	
											Pour les membres du ménage âgées de 15 ans et plus	Femme âgée de 15-49 ans				Pour tous les membres du ménage
HL1 N0. Lign e	HL2 RENOM ET NO	HL3.QUEL LIEN DE PARENTE ENTRE (PRENOM) ET LE CHEF DE MENAGE ?	HL4. DE QUEL SEXE (MASCULIN OU FEMININ) EST (NOM)? 1 Masculin 2 Féminin		HL5A. (PRENOM) RESIDE-T-IL HABITUELLEMENT DANS CE 1 Oui 2 Non		HL5. QUELLE EST LA DATE DE NAISSANCE DE (PRENOM)? 98 NSP 98 NSP 9998 NSP S'assurer que toutes les personnes de moins de 6 ans ont le mois et l'année de naissance renseignés.			QUEL ÂGE A (PRENOM)? Enregistrez en années révolues Si 95 ans ou plus, enregistrez '95'	HL7. QUELLE EST ACTUELLEMENT LA SITUATION MATRIMONIALE DE (PRENOM) ? 1 Marié ou en couple 2 Divorcé / Séparé 3 Veuf (ve) 4 Jamais marié ou en	HL8. Entourez le numéro de ligne s'il s'agit d'une femme âgée de 15-49 ans	HL9. Numéro de Ménage (M) de la femme	HL9A. Numéro de Ménage (M) de la mère de la femme Si la femme ne dépend pas de sa mère, enregistrez 00	HL10. Numéro d'identifiant de la femme	HL9. DEPUIS COMIEN D'ANNEES (PRENOM) VIT-IL/ELLE DANS CE Enregistrez 0 si moins d'une année
Ligne	Prénom et Non	Lien de parent	M	F	O	N	jj	mm	Année	Age	Situation matrimoniale	15-49				Années
11			1	2	1	2	___ ___	___ ___	__________		1 2 3 4	11	___ ___	___ ___	_ _ _ _ _ _ _ _	
12			1	2	1	2	___ ___	___ ___	__________		1 2 3 4	12	___ ___	___ ___	_ _ _ _ _ _ _ _	
13			1	2	1	2	___ ___	___ ___	__________		1 2 3 4	13	___ ___	___ ___	_ _ _ _ _ _ _ _	
14			1	2	1	2	___ ___	___ ___	__________		1 2 3 4	14	___ ___	___ ___	_ _ _ _ _ _ _ _	
15			1	2	1	2	___ ___	___ ___	__________		1 2 3 4	15	___ ___	___ ___	_ _ _ _ _ _ _ _	
16			1	2	1	2	___ ___	___ ___	__________		1 2 3 4	16	___ ___	___ ___	_ _ _ _ _ _ _ _	

Cochez si un questionnaire addit ☐

Demandez s'il y d'autres membres de la famille qui on été omis. Spécialement les enfants ou nourrissons qui ne sont pas sur la liste ci-dessus, et d'autres personnes qui ne sont pas membres de la famille tels les servantes ou amis mais qui vivent habituellement dans ce ménage.

Maintenant pour toutes les femmes âgées de 15-49 ans, Ecrire leur nom et numéro de ligne et autres identifiants sur la fiche information du questionnaire femme. Vous devriez maintenant avoir un questionnaire femme pour chaque femme éligible du ménage (âgée de 15-49 ans)

* Codes de HL3: Lien de parenté avec le chef de ménage: .

01 Chef de ménage	09 Beau-frère/ Belle-sœur	13 Adoption/enfants confiés
02 Epoux (se)	10 Oncle/Tante	14 Pas de lien
03 Fils/ Fille	11 Neveu/ Nièce	98 Ne sait pas
04 beau-fils/belle-fille	12 Autre relation	

**INFORMATION FEMMES**	**WM**
Ce questionnaire doit être administré à toutes les femmes âgées de 15 à 49 ans (voir Fiche de dénombrement des ménages, colonne HL8. Il faudra utiliser un pour chaque femme éligible.	
WM1A. DISTRICT<br>BARAOUELI .... 1<br>NIONO 2	WM1B. AIRES DE SANTE<br>MOLODO .... 1<br>NARA IBT .... 2<br>KALAKE .... 3<br>SANANDO 4
WM1C. Nom et Numéro du Village:<br>Nom: ______________________________ Numéro ______	WM1D. Nom et Numéro du Relais<br>Nom: ______________________ Numéro _______
WM2. Numéro de concession: R ___ ___ | C ___ ___	WM3. Numéro de ménage (de la base recensement)<br>___ ___ ___ ___ ___ ___ ___ ___ ___ ___ ___
WM4. Nom de la femme<br>Nom: ____	WM5. Numéro de ligne de la femme (HL1): _________________
WM6. Nom et Numéro l'enquêteur (trice):<br>Nom: ______________________________ Numéro ______	WM7. Jour/ Mois<br>________/______/2013

Lire le formulaire de consentement Femme au répondant du questionnaire Femme avant de poursuivre l'interview.

La permission est-elle accordée?

☐ *Oui, la permission est accordée* ➔ *Allez à WM10 pour enregistrer l'heure et ensuite démarrer l'interview.*

☐ *Non, la permission n'est pas accordée* ➔ *Complétez WM8. Discutez de ce résultat d'interview avec votre contrôleur*

WM8. Résultat de l'enquête Femme	
	Rempli: 01
	Pas a la maison 02
	Réfusé 03
	Rempli partiellement 04
	Incapacité 05
	Autres (*Précisez*): _________________________ 06

Notes de l'enquêteur (trice)

WM9 Contrôleur (Nom et Numéro): _______________________________________ _____________

WM10. Enregistrez l'heure.	Heures et minutes: ___ ___: ___ ___

ANTECEDENTS FEMMES		WB
WB1. EN QUEL MOIS ET EN QUELLE ANNEE ETES-VOUS NEE?<br>*Inserez '98 (pour mois), apres avoir insister pour avoir le mois de naissance*	Date de naissance<br>Mois [ ][ ]<br>Annee [ ][ ][ ][ ]	
WB2. QUEL AGE AVEZ-VOUS?<br>*Insistez* : QUEL AGE AVIEZ-VOUS A VOTRE DERNIER ANNIVERSAIRE?<br>*Comparez et corriger WB1 et/ou WB2 si incohérence*	Age (en annees completes) [ ][ ]	
WB3.AVEZ-VOUS FREQUENTE L'ECOLE CLASSIQUE OU MEDERSA?	Oui, école classique .... 1<br>Oui, medersa .... 2<br>Non 3	→ CM1
WB4. QUEL EST LE PLUS HAUT NIVEAU D'ETUDES QUE VOUS AVEZ ATTEINT?	Primaire (1 à 6) .... 1<br>Secondaire 1 (7 à 9) .... 2<br>Secondaire 2 (10 à 12) .... 3<br>Superieur 4	
WB5. QUELLE EST LA DERNIERE ANNEE / CLASSE QUE VOUS AVIEZ ACHEVE AVEC SUCCES A CE NIVEAU ?<br>*Si moins d'une année, enregistrez "00"*	Classe/Annee .... [ ][ ]	

MORTALITE INFANTILE CM

MAINTENANT, JE VOUDRAIS VOUS POSER DES QUESTIONS EN RAPPORT AVEC TOUTES LES GROSSESSES QUE VOUS AVEZ EU DANS VOTRE VIE. PAR CELA, JE VEUX PARLER DE TOUS LES ACCOUCHEMENTS QUE VOUS AVEZ EU, QUE CES DERNIERS SOIENT NES VIVANTS OU MORTS-NES, QU'ILS SOIENT ENCORE EN VIE OU PAS, ET AUSSI LES GROSSESSES QUE VOUS AVEZ EUES MAIS QUI NE SE SONT PAS ACHEVEES PAR DES NAISSANCES.

CM1. AVEZ-VOUS DEJA DONNE NAISSANCE A DES ENFANTS ?	Oui ...... 1<br>Non 2	2 ➔ CM8
CM2. AVEZ-VOUS DES FILS OU DES FILLES A QUI VOUS AVEZ DONNE NAISSANCE ET QUI VIVENT ACTUELLEMENT AVEC VOUS ?	Oui ...... 1<br>Non 2	2 ➔ CM4
CM3. COMBIEN DE FILS VIVENT AVEC VOUS ?<br>ET COMBIEN DE FILLES VIVENT AVEC VOUS ?<br>*Si aucun, inscrivez '00"*	Fils à la maison...... [ ][ ]<br>Filles à la maison...... [ ][ ]	
CM4. AVEZ-VOUS DES FILS OU FILLES A QUI VOUS AVEZ DONNE NAISSANCE QUI SONT TOUJOURS EN VIE MAIS QUI NE VIVENT PAS AVEC VOUS ?	Oui ...... 1<br>Non 2	2 ➔ CM6
CM5. COMBIEN DE FILS SONT VIVANTS MAIS QUI NE VIVENT PAS AVEC VOUS ?<br>COMBIEN DE FILLES SONT VIVANTES MAIS QUI NE VIVENT PAS AVEC VOUS ?<br>*Si aucun, inscrivez '00"*	Fils ailleurs ...... [ ][ ]<br>Filles ailleurs ...... [ ][ ]	
CM6. AVEZ-VOUS DEJA DONNE NAISSANCE A UN GARÇON OU A UNE FILLE QUI EST NE VIVANT MAIS QUI EST DECEDE PAR LA SUITE ?<br>*Si aucun, vérifiez en disant:*<br>JE VOUDRAIS DIRE, AUCUN BEBE QUI A RESPIRE, CRIE OU MONTRE UN SIGNE DE VIE - MEME S'IL N'A SURVECU QUE QULEQUES MINUTES?	Oui ...... 1<br>Non 2	2 ➔ CM8
CM7. COMBIEN DE GARÇONS SONT DÉCÉDÉS ?<br>COMBIEN DE FILLES SONT DECEDES ?<br>*Si aucun, inscrivez '00"*	Garcons décédés ...... [ ][ ]<br>Filles décédées ...... [ ][ ]	
CM8. DES GROSSESSES PEUVENT SE TERMINER PAR LA NAISSANCE D'UN BEBE QUI EST DEJA MORT, QU'ON APPELLE SOUVENT MORT-NE. AVEZ-VOUS DEJA ACCOUCHE D'UN MORT-NE?	Oui 1 ➔ *Demandez*<br>COMBIEN DE MORTS-NES? [ ][ ]<br>Non 2	
CM9. QUELQUES FOIS, DES FEMMES PORTENT DES GROSSESSES QUI NE SE TERMINENT PAS PAR DES NAISSANCES VIVANTES ET S'ARRETENT BIEN AVANT LA FIN (LE TERME) DE LA GROSSESSE, PAR EXEMPLE LES FAUSSES-COUCHES OU LES AVORTEMENTS. EN DEHORS DES MORTS-NES, AVEZ-VOUS DEJA EU DES FAUSSES-COUCHES OU DES AVORTEMENTS?	Oui 1 ➔ *Demandez*<br>AU TOTAL, COMBIEN DE FAUSSES-COUCHES ET AVORTEMENTS? [ ][ ]<br>Non 2	
CM10. FAITES LA SOMME DES REPONSES À CM3, CM5 ET CM7<br>*Si aucun, inscrivez '00"*	Somme (Naissances vivantes) [ ][ ]	
CM11. FAITES LA SOMME DES REPONSES À CM8 ET CM9<br>*Si aucun, inscrivez '00"*	Somme (Autres grossesses) [ ][ ]	

CM12. JE VOUDRAIS ETRE SUR QUE JE VOUS AI BIEN COMPRISE: VOUS AVIEZ EU AU TOTAL __________ (TOTAL A CM10) NAISSANCES VIVANTES DANS VOTRE VIE. EST-CE BIEN EXACT?

[ ] Oui [ ] Non- Insistez et corrigez CM3 a CM7 et CM10 si necessaire

CM13. ET VOUS AVIEZ EU AU TOTAL __________ (TOTAL A CM11) MORT-NES, FAUSSES-COUCHES ET AVORTEMENTS DANS VOTRE VIE. EST-CE BIEN EXACT?

[ ] Oui [ ] Non- Insistez et corrigez CM8, CM9 et CM11 si necessaire

CM14. VERIFIEZ TOTAL DE CM10 ET CM11

[ ] Une ou plusieurs grossesses ➔ PH1 (Section suivant) [ ] Pas de grossesse ➔ PH18

HISTORIQUE DE GROSSESSE — PH

MAINTENANT, JE VOUDRAIS ENREGISTRER TOUTES VOS GROSSESSES, QU'ELLES SE SOIENT TERMINEES PAR DES NAISSANCES VIVANTES, MORT-NES OU AVORTEMENT (FAUSSE-COUCHE).

COMMENCEZ S'IL VOUS PLAIT PAR LA PREMIERE GROSSESSE QUE VOUS AVIEZ EUE.

Engeristrez toutes les grossesses. Enregistrez les jumeaux et les triplets sur des lignes differentes

PH1	PH2	PH3	PH4	PH5	PH6	PH7	PH8
N0 de Ligne	VOTRE PREMIERE GROSSESSE/ GROSSESSE SUVANTE ETAIT-ELLE UNE GROSSESSE SIMPLE OU MULTIPLE (JUMEAUX)?	LE BEBE EST-IL NE VIVANT, MORT-NE, OU FAUSSE COUCHE (AVORTEMENT) ?	CE BEBE A-T-IL CRIE, BOUGE OU RESPIRE LORSQU'IL EST NE?	QUEL ETAIT LE NOM DONNE AU BEBE?	(PRENOM) EST-IL/ELLE DE SEXE MASCULIN OU FEMININ?	EN QUEL MOIS ET EN QUELLE ANNEE (PRENOM) EST-IL NE? Inserez '98' (pour mois), seulement si, incapable apres insistance d'obtenir le mois.	(PRENOM) EST-IL/ELLE ENCORE EN VIE?
01	Simple 1<br>Multiple 2	Ne (e) vivant (e) 1 → PH5<br>Mort-ne (e) 2<br>Fausse couche 3 → PH13	Oui 1<br>Non 2 → PH13	___________<br>___________<br>(Nom)	Garcon 1<br>Fille 2	Mois [ ][ ]<br>Annee [ ][ ][ ][ ]	Oui 1<br>Non 2 → PH12
02	Simple 1<br>Multiple 2	Ne (e) vivant (e) 1 → PH5<br>Mort-ne (e) 2<br>Fausse couche 3 → PH13	Oui 1<br>Non 2 → PH13	___________<br>___________<br>(Nom)	Garcon 1<br>Fille 2	Mois [ ][ ]<br>Annee [ ][ ][ ][ ]	Oui 1<br>Non 2 → PH12
03	Simple 1<br>Multiple 2	Ne (e) vivant (e) 1 → PH5<br>Mort-ne (e) 2<br>Fausse couche 3 → PH13	Oui 1<br>Non 2 → PH13	___________<br>___________<br>(Nom)	Garcon 1<br>Fille 2	Mois [ ][ ]<br>Annee [ ][ ][ ][ ]	Oui 1<br>Non 2 → PH12
04	Simple 1<br>Multiple 2	Ne (e) vivant (e) 1 → PH5<br>Mort-ne (e) 2<br>Fausse couche 3 → PH13	Oui 1<br>Non 2 → PH13	___________<br>___________<br>(Nom)	Garcon 1<br>Fille 2	Mois [ ][ ]<br>Annee [ ][ ][ ][ ]	Oui 1<br>Non 2 → PH12

Si ne (e) vivant (e) et est encore en vie			*Si ne (e) vivant (e) mais decede (e) maintenant*	*Si mort-ne (e) ou fausse couche /avortement*			
PH9	PH10	PH11	PH12	PH13	PH14	PH15	PH16
QUEL AGE AVAIT (PRENOM) A SON DERNIER ANNIVER-SAIRE? *Enregistrez age en annees completes*	(PRENOM) VIT-IL/ELLE AVEC VOUS?	Enregistrez le numero de ligne Menage de l'enfant (enregistrez '00' si enfant n'est pas sur la fiche denombrememt des menages)	QUEL AGE AVAIT-IL/ELLE AU DECES? Si '1an' insistez: COMBIEN DE MOIS AVAIT (PRENOM)? Enregistez jours si moins de 1 mois, et mois si moins de 2 ans, en annees	EN QUEL MOIS ET EN QUELLE ANNEE LA GROSSESE S'EST-ELLE ARRETEE? Inserez '98' (pour mois), seulement si, incapable apres insistance d'obtenir le mois.	COMBIEN DE MOIS LA GROSSESSE A-T-ELLE DURE? Enregistrez en mois complet	AVIEZ-VOUS PAR VOUS- MEME ARRETER CETTE GROSSESSE OU AVEC L'APPUI DE QUELQU'UN D'AUTRE ? Posez cette question seulement si PH3 a le code '3' entoure	Y-AVAIT-IL D'AUTRES GROSSESSES ENTRE CELLE-CI ET LA GROSSESSE ANTERIEURE?(ETES VOUS SUR DE N'AVOIR PAS OUBLIER UNE GROSSESSE ENTRE CELLES ANTERIEURES ET CELLE-CI?)
01<br>Age en annees	Oui 1<br>Non 2<br>→ *PH16*	N0 Ligne<br>→ *PH16*	J 1<br>M 2<br>A 3<br>→ *PH16*	Mois<br>Annee	Mois	Oui 1<br>Non 2	Oui 1<br>Non 2
02<br>Age en annees	Oui 1<br>Non 2<br>→ *PH16*	N0 Ligne<br>→ *PH16*	J 1<br>M 2<br>A 3<br>→ *PH16*	Mois<br>Annee	Mois	Oui 1<br>Non 2	Oui 1<br>Non 2
03<br>Age en annees	Oui 1<br>Non 2<br>→ *PH16*	N0 Ligne<br>→ *PH16*	J 1<br>M 2<br>A 3<br>→ *PH16*	Mois<br>Annee	Mois	Oui 1<br>Non 2	Oui 1<br>Non 2
04<br>Age en annees	Oui 1<br>Non 2<br>→ *PH16*	N0 Ligne<br>→ *PH16*	J 1<br>M 2<br>A 3<br>→ *PH16*	Mois<br>Annee	Mois	Oui 1<br>Non 2	Oui 1<br>Non 2
05<br>Age en annees	Oui 1<br>Non 2<br>→ *PH16*	N0 Ligne<br>→ *PH16*	J 1<br>M 2<br>A 3<br>→ *PH16*	Mois<br>Annee	Mois	Oui 1<br>Non 2	Oui 1<br>Non 2
06<br>Age en annees	Oui 1<br>Non 2<br>→ *PH16*	N0 Ligne<br>→ *PH16*	J 1<br>M 2<br>A 3<br>→ *PH16*	Mois<br>Annee	Mois	Oui 1<br>Non 2	Oui 1<br>Non 2
07<br>Age en annees	Oui 1	N0 Ligne	J 1<br>M 2	Mois<br>Annee	Mois	Oui 1	Oui 1

Non 2 → *PH16*	A 3 → *PH16*	→ *PH16*		Non 2	Non 2	

PH17. AVIEZ-VOUS EU UNE AUTRE GROSSESSE APRES LA DERNIERE QUE VOUS VENEZ DE MENTIONNER?<br><br>*Exclure grossesse actuelle*	Oui 1<br>Non 2	→ PH1
PH18. ETES-VOUS ENCEINTE PRESENTEMENT	Oui 1<br>Non 2<br>NSP 8	 → PH20<br>→ PH20
PH19. DE COMBIEN DE MOIS ETES-VOUS ENCEINTE?	☐☐ Mois	

PH20. Comptez le nombre de naissances vivantes en comptant les lignes <u>avec code PH6 entoure pour garcon ou fille</u>

Entrez total → ☐☐

Ce nombre que vous venez d'entrer est-il le meme que celui de CM10?

☐ *Oui* ☐ *Non* → *Insistez et faites les corrections necessaires*

PH21. Comptez le nombre des autres grossesses en comptant les lignes <u>sans code PH6 entoure pour garcon ou fille</u>

Entrez total → ☐☐

Ce nombre que vous venez d'entrer est-il le meme que celui de CM11?

☐ *Oui* ☐ *Non* → *Insistez et faites les corrections necessaires*

PH22. Revisez les items suivants dans la section Historique de Grossesses et cocher chaque case après verification

** Pour chaque grossesse, l'annee est enregistree dans PH7 ou PH13* ☐

** Pour chaque enfant vivant: l'age actuel est enregistre dans PH9* ☐

** Pour chaque enfant decede: l'age au deces est enregistre dans PH12* ☐

** Pour chaque enfant decede à l' age de 1 an (12 mois), insistez pour avoir le nombre exact de mois* ☐

HISTORIQUE DE GROSSESSE (CONTINUED)							PH
Ceci est la suite apres la Ligne 07 de la page precedente<br><br>*Engeristrez toutes les grossesses. Enregistrez les jumeaux et les triplets sur des lignes differentes*							
PH1	PH2	PH3	PH4	PH5	PH6	PH7	PH8
N0 de Ligne	VOTRE PREMIERE GROSSESSE/ GROSSESSE SUVANTE ETAIT-ELLE UNE GROSSESSE SIMPLE OU MULTIPLE (JUMEAUX)?	LE BEBE EST-IL NE VIVANT, MORT-NE, OU MORT AVANT LA FIN (LE TERME) DE LA GROSSESSE?	CE BEBE A-T-IL CRIE, BOUGE OU RESPIRE LORSQU'IL EST NE?	QUEL ETAIT LE NOM DONNE AU BEBE?	(PRENOM) EST-IL/ELLE DE SEXE MASCHULIN OU FEMININ?	EN QUEL MOIS ET EN QUELLE ANNEE (PRENOM) EST-IL NE? Inserez '98' (pour mois), seulement si, incapable apres insistance d'obtenir le mois.	(PRENOM) EST-IL/ELLE ENCORE EN VIE?
08	Simple 1<br>Multiple 2	Ne (e) vivant (e) 1 → PH5<br>Mort-ne (e) 2<br>Mort avant terme 3 → PH13	Oui 1<br>Non 2 → PH13	___________<br>___________<br>(Nom)	Garcon 1<br>Fille 2	Mois [ ][ ]<br>Annee [ ][ ][ ][ ]	Oui 1<br>Non 2 → PH12
09	Simple 1<br>Multiple 2	Ne (e) vivant (e) 1 → PH5<br>Mort-ne (e) 2<br>Mort avant terme 3 → PH13	Oui 1<br>Non 2 → PH13	___________<br>___________<br>(Nom)	Garcon 1<br>Fille 2	Mois [ ][ ]<br>Annee [ ][ ][ ][ ]	Oui 1<br>Non 2 → PH12
10	Simple 1<br>Multiple 2	Ne (e) vivant (e) 1 → PH5<br>Mort-ne (e) 2<br>Mort avant terme 3 → PH13	Oui 1<br>Non 2 → PH13	___________<br>___________<br>(Nom)	Garcon 1<br>Fille 2	Mois [ ][ ]<br>Annee [ ][ ][ ][ ]	Oui 1<br>Non 2 → PH12
11	Simple 1<br>Multiple 2	Ne (e) vivant (e) 1 → PH5<br>Mort-ne (e) 2<br>Mort avant terme 3 → PH13	Oui 1<br>Non 2 → PH13	___________<br>___________<br>(Nom)	Garcon 1<br>Fille 2	Mois [ ][ ]<br>Annee [ ][ ][ ][ ]	Oui 1<br>Non 2 → PH12

Si ne (e) vivant (e) et est encore en vie			*Si ne (e) vivant (e) mais decede (e) maintenant*	*Si mort-ne (e) ou mort avant la naissance*			
PH9	PH10	PH11	PH12	PH13	PH14	PH15	PH16
QUEL AGE AVAIT (PRENOM) A SON DERNIER ANNIVER-SAIRE? *Enregistrez l'age en annees completes*	(PRENOM) VIT-IL/ELLE AVEC VOUS?	Enregistrez le numero de ligne Menage de l'enfant (enregistrez '00' si enfant n'est pas sur la fiche denombremenmt des menages)	QUEL AGE AVAIT-IL/ELLE AU DECES? Si '1an' insistez: COMBIEN DE MOIS AVAIT (PRENOM)? Enregistez jours si moins de 1 mois, et mois si moins de 2 ans, en annees	EN QUEL MOIS ET EN QUELLE ANNEE LA GROSSESE S'EST-ELLE ARRETEE? Inserez '98' (pour mois), seulement si, incapable apres insistance d'obtenir le mois.	COMBIEN DE MOIS LA GROSSESSE A-T-ELLE DURE? Enregistrez en mois complet	AVIEZ-VOUS PAR VOUS- MEME OU QUELQU'UN A-T-IL FAIT QUELQUE CHOSE POUR ARRETER CETTE GROSSESSE? Posez cette question seulement si PH3 a le code '3' entoure	Y-AVAIT-IL D'AUTRES GROSSES SES ENTRE CELLES ANTERIEU RES ET CELLE-CI?
08<br>Age en annees	Oui 1<br>Non 2<br>→ *PH16*	N0 Ligne<br>→ *PH16*	J 1<br>M 2<br>A 3<br>→ *PH16*	Mois<br>Annee	Mois	Oui 1<br>Non 2	Oui 1<br>Non 1
09<br>Age en annees	Oui 1<br>Non 2<br>→ *PH16*	N0 Ligne<br>→ *PH16*	J 1<br>M 2<br>A 3<br>→ *PH16*	Mois<br>Annee	Mois	Oui 1<br>Non 2	Oui 1<br>Non 1
10<br>Age en annees	Oui 1<br>Non 2<br>→ *PH16*	N0 Ligne<br>→ *PH16*	J 1<br>M 2<br>A 3<br>→ *PH16*	Mois<br>Annee	Mois	Oui 1<br>Non 2	Oui 1<br>Non 1
11<br>Age en annees	Oui 1<br>Non 2<br>→ *PH16*	N0 Ligne<br>→ *PH16*	J 1<br>M 2<br>A 3<br>→ *PH16*	Mois<br>Annee	Mois	Oui 1<br>Non 2	Oui 1<br>Non 1
12<br>Age en annees	Oui 1<br>Non 2<br>→ *PH16*	N0 Ligne<br>→ *PH16*	J 1<br>M 2<br>A 3<br>→ *PH16*	Mois<br>Annee	Mois	Oui 1<br>Non 2	Oui 1<br>Non 1
13<br>Age en annees	Oui 1<br>Non 2<br>→ *PH16*	N0 Ligne<br>→ *PH16*	J 1<br>M 2<br>A 3<br>→ *PH16*	Mois<br>Annee	Mois	Oui 1<br>Non 2	Oui 1<br>Non 1
14<br>Age en annees	Oui 1	N0 Ligne	J 1<br>M 2	Mois<br>Annee	Mois	Oui 1	Oui 1

Non 2		A 3							
→ *PH16*	→ *PH16*	→ *PH16*			Non 2	Non 1			

OBSERVATIONS DE L'ENQUETEUR/ L'ENQUÊTRICE

OBSERVATION CONTROLEUR

OBSERVATION DU SUPERVISEUR
